# Factors Associated with Increased Risk Perception of Pandemic Influenza in Australia

**DOI:** 10.1155/2010/947906

**Published:** 2010-07-20

**Authors:** Jennifer Jacobs, Melanie Taylor, Kingsley Agho, Garry Stevens, Margo Barr, Beverley Raphael

**Affiliations:** ^1^School of Medicine, University of Western Sydney, Penrith South DC, NSW 1797, Australia; ^2^New South Wales Department of Health, Centre for Epidemiology and Research, NSW 2059, Australia

## Abstract

The aim of this study was to assess factors associated with increased risk perception of pandemic influenza in Australia. The sample consisted of 2081 Australian adults aged 16 years and older who completed a short three item pandemic influenza question module which was incorporated into the NSW Health Adult Population Health Survey during the first quarter of 2007. After adjusting for covariates, multivariate analysis indicated that those living in rural regions were significantly more likely to perceive a high risk that a pandemic influenza would occur, while those with poor self-rated health perceived both a high likelihood of pandemic and high concern that self/family would be directly affected were such an event to occur. Those who spoke a language other than English at home and those on low incomes and younger people (16–24 years) were significantly more likely to have changed the way they lived their lives due to the possibility of pandemic influenza, compared to those who spoke only English at home, middle-high income earners, and older age groups, respectively. This data provides an Australian population baseline against which the risk perceptions of demographic subgroups regarding the current, and potential future pandemics, can be compared and monitored.

## 1. Introduction

The pattern of recurrence of pandemics since the mid-eighteenth century indicates that pandemics occur about every 30 years [1]. Prior to 2009, expert consensus was that another pandemic influenza was almost inevitable [2–7], and although the H5N1 avian viruses were the most likely candidate for an influenza outbreak, the unexpected H1N1 swine influenza reached pandemic in June, 2009. With previous influenza pandemics and the current H1N1 influenza pandemic arriving with little to no warning, we are afforded a unique opportunity to prepare for the next pandemic threat, which has the potential to be more severe than the current pandemic. Important for preparation is knowledge about the public's response to such a threat, and a key component to the public's response is their perception of risk.

Knowing how a risk is perceived is essential for preparing an effective plan for risk communication, and may be predictive of the public's response. In a study of the NSW population, Barr et al. [8] found that respondents with higher levels of risk perception reported more willingness to comply with public health behaviours in the event of an outbreak of influenza. Similar results were found in Hong Kong [9] and Italy [10], where respondents in both studies with an increased perception of risk were more likely to be engaged in risk-reducing behaviours.

In 2007, 14.9% of the NSW population reported that they thought pandemic influenza was very or extremely likely to occur and 45.5% were very or extremely concerned that they or their family would be affected by an influenza pandemic should it occur [8]. What may be of particular importance however, is how risk perception varies within the population. Risk perception may be affected by factors such as awareness of a hazard, cultural and social factors or the experience or memory of a prior similar hazard, all of which may result in variation in risk perception among individuals. Lau et al. [11] found in a Hong Kong sample that the odds of females reporting worry about themselves or their families contracting an outbreak of avian influenza if it is to occurr were 1.6 times higher than the odds of males reporting such worry. De Zwart et al. [12] similarly found that women and older respondents scored significantly higher on a composite measure of risk perception (combining perceived seriousness of threat and vulnerability to threat) than men and younger respondents, respectively. In an Italian population, Di Giuseppe et al. [10] found that risk perception was higher for respondents with lower socioeconomic status and lower education.

In preparation for a pandemic influenza outbreak, the Australian Government recommends a number of measures the general public could take, such as having enough food, water, and essential items to enable a household to be confined at home for up to 14 days [13], ensuring such food is rotated and use by dates are checked regularly [13]; practicing good personal hygiene, and teaching children about hand washing and cough etiquette [14].The World Health Organisation has also recommended seasonal influenza vaccinations for health care workers to reduce the risk of genetic shifts in the influenza virus [15].The preparation of the general public for an outbreak of influenza may be a key strategy in preventing the spread of the disease in the event of a pandemic. Thus it is important to identify subpopulations in Australia who are more and less likely to have changed their life in response to the possibility of pandemic influenza.

The aim of the current study was to obtain baseline Australian data on factors associated with perceptions of the likelihood of pandemic influenza, concern for self and family in the event of an influenza pandemic and broad changes in living as a result of the threat of pandemic influenza.

## 2. Methods

A short three item pandemic influenza question module was developed as the first part of a larger module of questions on potential threats. These questions were field tested and inserted into the New South Wales Population Health Survey, administered between 22 January and 31 March, 2007 [8]. The New South Wales Population Health Survey is a continuous telephone survey including questions on health behaviours, health status, and access to health services of the state population using the in-house CATI facility of the New South Wales Department of Health [16]. Households were contacted using random digit dialing. Up to 7 calls were made to establish initial contact with a household, and 5 calls were made in order to contact a selected respondent. Only residential phone numbers were used in the sample, as residential phone coverage in Australia still remains high [17] and results from persons who only have mobile phones has been shown to be comparable in the United States [18, 19]. Interviews were conducted in English, Arabic, Chinese, Greek, Italian, or Vietnamese, depending on respondent preference. More details of the sampling approach can be found in the 2007 NSW Health survey report [20].

### 2.1. Question Module

A three item pandemic influenza question module was developed which addressed pandemic influenza threat perceptions. The wording of the questions was as follows:

1. How likely do you think it is that pandemic influenza will occur in Australia?
2. If a pandemic influenza were to occur in Australia, how concerned would you be that you or your family would be affected by it?
3. How much have you changed the way you live your life because of the possibility of an influenza pandemic?

All responses were coded on a five-point Likert scale. Response options for all questions were “not at all”, “a little”, “moderately”, “very”, and “extremely”. In addition, “do not know” and “refused” responses were coded as missing.

### 2.2. Data Analysis

Data analysis was performed using the “SVY” commands of Stata version 9.2 (Stata Corp, College Station, TX, USA), which allowed for adjustments for sampling weights. 

The five-point Likert-scale responses were dichotomised. The definitions of the variables used are as follows:

1. *Pandemic influenza likely to occur:* the proportion of households aged 16 years and older who rated pandemic influenza as very or extremely likely to occur.
2. *Concern for self/family:* the proportion of households aged 16 years and older who were very or extremely concerned that self/family would be directly affected if pandemic influenza were to occur.
3. *Changed way of living:* the proportion of households aged 16 years and older who had changed the way they live their life a little, moderately, very or extremely because of the possibility of an influenza pandemic.
4. *Combined indicator* (1): *pandemic influenza likely to occur* + Concern for self/family
5. *Combined indicator* (2): *pandemic influenza likely to occur* + Concern for self/family + Changed way of living.

To determine factors associated with risk perception, the dichotomized risk question indicators and the “composite” indicators were used as outcome measures and these were investigated using the following set of independent variables: age, gender, marital status, children in household, location (urban/rural) as defined by respondents' area health region, born in Australia, speaking a language other than English at home, highest level of formal education, household income, living alone, self-rated health status, and psychological distress. Self-rated health status was assessed with the question “Overall, how would you rate your health during the past 4 weeks?” with possible responses being “excellent”, “very good”, “good”, “fair”, “poor”, and “very poor”. Responses of “very good” and “good” were combined and reported as “good” self-rated health, and responses of “poor” and “very poor” were combined and reported as “poor” self-rated health. Psychological distress was assessed using the Kessler 10 measure (K10). The K10 provides a measure of nonspecific psychological distress. Questions in the K10 include “In the past 4 weeks about how often did you feel …” “tired out for no good reason”, “nervous”, “so nervous that nothing could calm you down”, “hopeless”, “restless of fidgety”, “so restless you could not sit still”, “depressed”, “that everything was an effort”, “so sad that nothing could cheer you up”, and “worthless”. Possible responses were “all of the time”, “most of the time”, “some of the time”, “a little of the time”, and “none of the time”. The K10 provides a score ranging from 10–50. For the current analysis a score below 22 was considered as low-psychological distress, and a score of 22 or above was considered as high-psychological distress.

Multiple survey logistic regression using stepwise backwards model was used in order to identify the factors significantly associated with risk perception. All variables with statistical significance of *P* ≤ .05 were retained in the final model.

## 3. Results

In total, 2081 state residents aged 16 and over completed the module on pandemic influenza. The overall response rate was 65%. The key demographics of the weighted survey were comparable to Australian Bureau of Statistics (ABS) 2006 Australian population census data [21].

### 3.1. Multivariate Analyses

Multiple survey logistic regression analyses using a backward stepwise method were performed for the nine outcome variables.  Table 1 shows that Australian households who lived in rural areas were significantly more likely to think that pandemic influenza was very or extremely likely to occur than those in urban region (AOR = 1.59 (95% CI: 1.02–2.49, *P* = .041)). Respondents with poor self-rated health were also significantly more likely to think that pandemic influenza was very or extremely likely to occur, compared to those with good self-rated health (AOR = 1.92 (95% CI: 1.12–3.31, *P* = .018)) and were also more likely to report being very or extremely concerned that self or family would be directly affected if a pandemic was to occur (AOR = 1.64 (95% CI: 1.09–2.47, *P* = .017). Those from low income households, those who spoke a language other than English, and young people (16–24 years) were more likely to have changed the way they lived their lives because of the possibility of pandemic influenza, compared to their respective reference groups.

Table 1 also shows that respondents who lived in rural areas and respondents who reported poor self-rated health were significantly more likely to report combined indicator (1) than those who lived in urban areas and those with good self-rated health. The odds of respondents with high-psychological distress reporting combined indicator (2) were 3.03 (AOR = 3.03) times higher than the odds of respondents with low-psychological distress levels reporting combined indicator (2).

## 4. Discussion

The aim of this study was to assess factors associated with increased risk perception of pandemic influenza in Australia, increased concern for self and family if a pandemic influenza were to occur in Australia and associated changes in living due to the threat of such an event. Particular strengths of this study are the population-based sampling method and appropriate adjustment for sampling weight to reflect the population of interest.

Generally, pandemic influenza was not regarded as a high threat by NSW residents, with only 14.9% reporting that they felt pandemic influenza was very or extremely likely to occur. Those living in rural areas and those with poor self-rated health were more likely to report pandemic influenza very or extremely likely to occur compared with those living in urban areas and those with good self-rated health, respectively. Although not regarded as a high threat by Australians, 45.5% of respondents said they would be very or extremely concerned for self and family in the event of a pandemic influenza. Respondents with poor self-rated health were more likely to report more concern for self and family if an influenza pandemic occurred as compared to respondents with good self-rated health.

These results are dissimilar to those in prior studies. Although the studies of Lau et al. [11] and De Zwart et al. [12] found that females scored higher on risk perception than males, gender was not a significant risk factor for high-perceived pandemic likelihood or concern for self and family in this study. Similarly, although in prior studies older respondents [12], those with lower socioeconomic status [10] and lower education [10] reported significantly higher risk perception, in the current study none of these were risk factors for high-perceived pandemic likelihood or concern for self and family. What is common to all these groups is that they represent the groups typically most vulnerable to concern due to a focal threat. The prior studies were conducted on populations responding to a tangible threat as they were conducted around either the time of the avian influenza outbreaks or on populations which were most affected by the avian influenza or the SARS outbreaks. As this study was conducted on an Australian population which was not directly affected by avian influenza or SARS and where influenza was not a media focus, the null findings in this study may indicate that the threat of pandemic influenza was so general and distal that it did not have the capacity to concern even the portion of the population normally most sensitive to threat.

It is not surprising that individuals with poor self-rated health reported greater risk perception and concern for self and others than individuals with good self-rated health. The health concerns of these individuals may lead them to believe they are more susceptible to infection or complications which may occur with an outbreak of pandemic influenza. Also, since poor self-rated health has been associated with increased levels of anxiety [22, 23] and distress [24], there may be a heightened focus on, concern about, and belief in the likelihood of major external threats such as a pandemic influenza would represent. Such anxiety and distress may also lead these individuals to be more concerned about others as well as themselves in the event of a pandemic influenza. The potential link between self-rated health and heightened risk perception and concern for self and other in the event of a risky external event such as pandemic influenza warrants further examination.

We can only speculate as to why individuals living in rural areas believed pandemic influenza was more likely to occur than those living in urban areas. Perhaps individuals living in rural areas are more broadly aware of disease transmission and its health and economic consequences, including the possibility of influenza transmission from animals to human. However, though individuals living in rural areas believed that pandemic influenza was more likely to occur than individuals living in urban areas, they did not display more concern for self and family should a pandemic influenza occur. This is not unexpected given that the influenza virus is more easily transmitted from person to person in crowded environments, and that rural environments are typically not densely populated. It might be expected however that individuals living in urban environments may be more concerned for self and family in the case of a pandemic influenza, as urban environments are typically crowded. This, however, was not the case in this study. Similarly, it is of particular note that in this study concern for self and family did not increase when there were children or elderly in the household, despite individuals in these age groups being particularly vulnerable to influenza morbidity and mortality. As suggested above, these null results might reflect that pandemic influenza is too distal a threat for concern for the whole Australian population. Perhaps higher levels of perceived likelihood and concern for self and family in the context of a specific imminent threat (e.g., swine flu) are required for significant group differentiation to emerge.

Generally, a minority of people had changed the way they live their life because of the possibility of pandemic influenza, with only 23.8% reporting they had changed their life at all. This is not surprising as the current data also indicate that few Australians believed pandemic influenza was likely to occur. Households which had a lower income, households which spoke a language other than English and those respondents aged between 16 and 24 were more likely to have changed the way they lived their life because of the possibility of a pandemic influenza than those households with middle-high income, those who only spoke English and those older than 24 years, respectively. Further investigation into specific actions people take to change their lives in response to the threat of a pandemic influenza may provide useful information.

Interestingly, all of the factors associated with living changes in the case of a pandemic influenza are independent of the factors associated with perceived likelihood and concern for self and family in the case of pandemic influenza. That is, these groups are reporting living changes in the absence of heightened perceived threat or concern relative to the remainder of the population. This suggests that these groups may not be changing their way of life because they feel pandemic influenza is more likely than the remainder of the population, or because they feel themselves or their families are particularly vulnerable should pandemic influenza occur, but for some other reason. It is possible that these results may be due to methodological issues. These groups (lower income, language other than English, and younger respondents) are somewhat marginalized groups, that we would expect to have a higher threat perception and concern. The results therefore may be due to the broad nature of the question, which may have tapped into a more general and pervasive sense of threat vulnerability within the community, such as upswings in terrorism, war, and climate change, which may have been felt more strongly in more exposed or vulnerable groups. Similarly, the response set for this question was extremely broad compared to the remaining questions. A respondent was considered to have changed his way of life if he reported to have done so a “little”, “moderately”, “very”, or “extremely”. This is in contrast to the perception and concern questions where only responses of “very” and “extremely” were included in analyses. Changed life a “little” could be interpreted by some respondents as an increased feeling of threat which represents a change in effect rather than in behaviour.

Even though individuals with poor self-rated health believed that pandemic influenza was more likely to occur and felt more concern for self and family in the case of a pandemic influenza than those with good self-rated health, they were not more likely to report changing their life as a result of the possibility of pandemic influenza. Again, these populations may be responding to a more distal than proximal threat. Though they report some concern, it may not have been of the extent to prompt actual changes in way of living. More practically, neither the government nor the media were concerned at this time with promoting that the population makes life changes as a result of the threat of pandemic influenza, nor what these actions should be.

In a previous study, the role of concern for self and family was a key factor associated with likely compliance with protective health behaviours [25]. This suggests the benefit of risk communication messages that strategically heighten and then utilise public concern when a pandemic has or is likely to occur to increase compliance behaviours. For example, risk communication strategies could selectively target sub-population for whom risk beliefs are particularly low; in the current study these groups are urban populations and populations with good self-rated health. However, some authors have cautioned that increasing the risk perception of the population through such strategies risks societal estrangement and may frighten health care workers, first responders, and those who would have contact with the public in the event of a pandemic [26]. When consensus is reached regarding the optimal level of risk perception required for specific populations to elicit appropriate protective responses, the results of this study may be useful to guide which population groups these artificially inflating or deflating risk communication messages should be targeted.

It is likely that due to the recent H1N1 swine influenza pandemic that the current risk perceptions of the population are significantly different to those reported in this paper. Further research could examine changes in risk perceptions following this current pandemic for the whole population as well as the subpopulations examined in this paper. As such, the results of this paper provide a baseline measure for which future studies on risk perceptions can be compared. The population may have also recently made changes in daily living as a result of the H1N1 pandemic, as information on preventative measures such as personal hygiene have featured prominently in social marketing messages since its outbreak. This represents a response to a pandemic rather than a preventative measure for a potential pandemic. However, it would be important to determine which subpopulations maintain key behaviours (e.g., sneeze etiquette) following the end of the current pandemic, as this information can assist in the prevention of future pandemic threats.

With the outbreak of the current H1N1 swine influenza, research has emerged which has reexamined pandemic influenza attitudes and reactions in the Australian population. In research conducted during the World Health Organization (WHO) Phase 5 of the swine flu pandemic (between 2 May and 29 May, 2009) [27], 21% of a Sydney-based sample ranked their risk of catching pandemic influenza as high. The same authors conducted a similar survey also on a Sydney-based population during the WHO Phase 6 of the swine flu pandemic (between September and October, 2009) and found that 17.4% believed they had a high to very high risk of acquiring H1N1 influenza [28]. In a CATI survey conducted between August and September, 2009, in an Australian nationally representative sample, Eastwood et al. [29] found that of the respondents, 5% were extremely concerned and 17% were quite concerned that they or a member of their family would contract swine influenza. Although in the latter study consideration was given to the reasons for concern (e.g., close family member/friend in high-risk group, having an underlying illness, being employed in a position with high public contact), none of these studies examined factors associated with increased perceived risk for acquiring swine H1N1 influenza. These results provide interesting comparison to the results of the current study. When the likelihood of pandemic influenza occurring was generally considered to be low, 45.5% of the population reported they would be concerned for themselves and their family should it occur. However, in the midst of the current pandemic, individuals perceived less risk to the self which has decreased as the influenza pandemic has progressed [27, 28], and with as few as 22% [29] of the population reporting concern that they or a family member would contract the virus.

Lastly, consideration should be given to the limitations of the current study. The main limitation is that the study was conducted using telephone interviews which may have introduced selection bias. However, residential phone coverage in Australia remains high [17], and a large number of studies on SARS and avian influenza have utilized this method. Also, risk perception and protective behaviours are likely to be mediated by a number of factors in addition to those identified in this study. Factors such as anxiety, risk perception of others, media, and recent events such as the current swine flu pandemic are all factors likely to affect risk perception. However, despite these limitations the results of this study suggest that it may be appropriate to direct risk communication strategies to individuals living in urban populations and individuals with good self-rated health, which may result in an increased likelihood of appropriate protective responses if an influenza pandemic was to occur. Data in this study further suggest that in contexts where pandemic influenza is generally not regarded as a high threat by the population, messages highlighting actions individuals can take to prepare for a pandemic influenza should be directed to households with higher incomes, households which do not speak a language other than English, and individuals above the age of 24 years. Finally, data from this study provide an Australian population baseline against which factors associated with risk perception related to outbreaks of pandemic influenza, both current and future, can be compared.

##  Competing Interests

The authors declared that there are no competing interests.

## Figures and Tables

**Table 1:** Adjusted odds ratio (95% confidence interval) for Pandemic Influenza likely, Concern for self/family, Changed life, combined indicator (1) and combined indicator (2).

Outcome variables	Independent variable	AOR (95% CI)	*P*-value
Pandemic Influenza likely	Location		
	Urban	1.00	
	Rural	1.59 (1.02, 2.49)	.041
	Health self-rated as good		
	Yes	1.00	
	No	1.92 (1.12, 3.31)	.018

Concern self or family directly affected	Health self-rated as good		
	Yes	1.00	
	No	1.64 (1.09, 2.47)	.017

Changed way of living	Age in categories		
	16–24	1.00	
	25–34	0.37 (0.17, 0.83)	.016
	35–44	0.51 (0.23, 1.10)	.085
	45–54	0.32 (0.15, 0.72)	.006
	55–64	0.24 (0.10, 0.57)	.001
	65–74	0.28 (0.12, 0.67)	.004
	75+	0.14 (0.05, 0.37)	<.001
	Speak language other than English		
	No	1.00	
	Yes	1.71 (1.05, 2.78)	.031
	Household income		
	<$20k	1.00	
	$20–$40k	0.71 (0.43, 1.16)	.170
	$40–$60k	0.66 (0.38, 1.14)	.137
	$60–$80k	0.45 (0.22, 0.91)	.027
	>$80k	0.50 (0.28, 0.87)	.015
	Marital status		
	Married	1.00	
	Widowed	1.27 (0.76, 2.13)	.357
	separated/divorced	1.02 (0.62, 1.66)	.951
	Never married	0.44 (0.26, 0.75)	.003

Pandemic influenza likely + concerned for self/family	Location		
	Urban	1.00	
	Rural	1.84 (1.03, 3.27)	.038
	Health self-rated as good		
	Yes	1.00	
	No	2.45 (1.28, 4.71)	.007

Pandemic influenza likely + concerned + changed way of living	Household income		
	<$20k	1.00	
	$20–$40k	1.00 (0.35, 2.85)	.997
	$40–$60k	0.23 (0.06, 0.84)	.026
	$60–$80k	0.63 (0.18, 2.21)	.465
	>$80k	0.54 (0.18, 1.61)	.268
	High Psychological distress		
	No	1.00	
	Yes	3.03 (1.13, 8.11)	.028

Note: Independent variables adjusted for are; age, marital status; have children less than 16 years; location (urban/rural); born in Australia; speak a language other than English at home; highest educational qualification; household income, self-rated health status and psychological distress (K10).

## References

[1] Simonsen L (1999). The global impact of influenza on morbidity and mortality. *Vaccine*.

[2] Beigel JH, Farrar J, Han AM (2005). Avian influenza A (H5N1) infection in humans. *New England Journal of Medicine*.

[3] Monto AS (2005). The threat of an avian influenza pandemic. *New England Journal of Medicine*.

[4] Osterholm MT (2005). Preparing for the next pandemic. *New England Journal of Medicine*.

[5] Perez DR, Sorrell EM, Donis RO (2005). Avian influenza: an omnipresent pandemic threat. *Pediatric Infectious Disease Journal*.

[6] Ritz N, Curtis N (2008). What the paediatrician needs to know when pandemic influenza arrives in clinical practice. *Advances in Experimental Medicine and Biology*.

[7] WHO (2004). World is ill-prepared for "inevitable" flu pandemic. *Bulletin of the World Health Organization*.

[8] Barr M, Raphael B, Taylor M (2008). Pandemic influenza in Australia: using telephone surveys to measure perceptions of threat and willingness to comply. *BMC Infectious Diseases*.

[9] Leung GM, Lam T-H, Ho L-M (2003). The impact of community psychological responses on outbreak control for severe acute respiratory syndrome in Hong Kong. *Journal of Epidemiology and Community Health*.

[10] Di Giuseppe G, Abbate R, Albano L, Marinelli P, Angelillo IF (2008). A survey of knowledge, attitudes and practices towards avian influenza in an adult population of Italy. *BMC Infectious Diseases*.

[11] Lau JTF, Kim JH, Tsui H, Griffiths S (2007). Anticipated and current preventive behaviors in response to an anticipated human-to-human H5N1 epidemic in the Hong Kong Chinese general population. *BMC Infectious Diseases*.

[12] de Zwart O, Veldhuijzen IK, Elam G (2007). Avian influenza risk perception, Europe and Asia. *Emerging Infectious Diseases*.

[13] Emergency Management Australia Preparing for an Emergency—the smart thing to do. http://www.pantrylist.com.au/pantry/dloads/PantryList.pdf.

[14] Australian Government About Pandemic Influenza, Protecting Yourself and Others. http://www.flupandemic.gov.au/internet/panflu/publishing.nsf/Content/protecting-all-1.

[15] http://www.whopak.org/pdf/Guidelines_for_bird_flu_Jan_2006.pdf.

[16] Barr M, Baker D, Gorringe M, Fritsche L Description of Methods. http://www.health.nsw.gov.au/resources/publichealth/surveys/health_survey_method.asp.

[17] Australian Bureau of Statistics Population Survey Monitor: Catalogue no 4103.0.

[18] Brick M, Dipko S, Presser S, Tucker C, Yuan Y (2005). Estimation Issues in Dual Frame Sample of Cell and Landline Numbers; ASA Section on Survey Research Methods.

[19] Yuan Y, Allen B, Brick MJ Surveying households on cell phones: results and lessons.

[20] Centre for Epidemiology and Research (2008). *2007 Report on Adult Health from the New South Wales Population Health Survey*.

[21] Australian Bureau of Statistics (2006). *Census of Population and Housing*.

[22] Public Health Division (2001). *Report on the 1997 and 1998 NSW Health Surveys*.

[23] Liavaag AH, Dørum A, Fosså SD, Tropé C, Dahl AA (2009). Morbidity associated with "self-rated health" in epithelial ovarian cancer survivors. *BMC Cancer*.

[24] Lima-Costa MF, Firmo JO, Uchôa E (2005). Differences in self-rated health among older adults according to socioeconomic circumstances: the Bambuí Health and Aging Study. *Cadernos de Saúde Pública*.

[25] Taylor M, Raphael B, Barr M, Agho K, Stevens G, Jorm L (2009). Public health measures during an anticipated influenza pandemic: factors influencing willingness to comply. *Risk Management and Health Care Policy*.

[26] Middaugh JP (2008). Pandemic influenza preparedness and community resiliency. *Journal of the American Medical Association*.

[27] Seale H, McLaws M-L, Heywood AE (2009). The community's attitude towards swine flu and pandemic influenza. *Medical Journal of Australia*.

[28] Seale H, Heywood AE, McLaws M-L (2010). Why do I need it? I am not at risk! Public perceptions towards the pandemic (H1N1) 2009 vaccine. *BMC Infectious Diseases*.

[29] Eastwood K, Durrheim DN, Jones A, Butler M (2010). Acceptance of pandemic (H1N1) 2009 influenza vaccination by the Australian public. *Medical Journal of Australia*.

